# Yeast β-glucan selenium nanoparticles enhance meat quality in heat-stressed broilers via SelO-mediated mitochondrial biogenesis and oxidative myofiber remodeling

**DOI:** 10.1186/s40104-026-01437-4

**Published:** 2026-07-04

**Authors:** Weiguang Yang, Chengkun Fang, Jiani Yang, Jing Lv, Guanyu Shang, Xinhao Song, Yujing Tang, Shusong Wu, Rejun Fang

**Affiliations:** 1 College of Animal Science and Technology, Hunan Agricultural University, Changsha, 410128 China; 2 Hunan Engineering Research Center of Intelligent Animal Husbandry, Changsha, 410128 China; 3 Yuelushan Laboratory, Changsha, 410128 China

**Keywords:** Heat stress, Meat quality, Mitochondrial function, Muscle fiber transformation, Selenium nanoparticles

## Abstract

**Background:**

Heat stress (HS) markedly impairs broiler growth, muscle function, and meat quality. In this study, broilers were subjected to a 2 × 2 factorial design with sodium selenite or yeast β-glucan selenium nanoparticles (yeast β-Glu-SeNPs) as the selenium source (0.3 mg/kg total selenium) under thermoneutral or HS conditions. We aimed to investigate the protective effects and underlying mechanisms of yeast β-Glu-SeNPs against HS-induced muscle damage.

**Results:**

HS markedly impaired growth performance and induced systemic oxidative stress and inflammation, while also compromising meat quality and disrupting postmortem glycolysis, as evidenced by reduced glycogen availability and excessive lactate accumulation. Yeast β-Glu-SeNPs significantly improved growth performance, mitigated oxidative stress and inflammation, and restored meat quality in both breast and thigh muscles. Postmortem energy metabolism was preserved, as reflected by increased muscle glycogen and glycolytic potential, reduced lactate accumulation and glycolytic enzyme activities, and a stabilized pH decline. Meanwhile, skeletal muscle Se deposition, glutathione peroxidase activity, and key selenoprotein expression were markedly enhanced. Notably, HS promoted a phenotypic shift toward fast glycolytic muscle fibers, as evidenced by increased expression of *MYHC2b* and Fast-MyHC (*P* < 0.05), accompanied by reduced levels of *MYHC1*, *MYHC2a*, and Slow-MyHC (*P* < 0.05). This maladaptive transition was effectively reversed by yeast β-Glu-SeNPs, which favored oxidative fiber formation, characterized by the upregulation of *MYHC1* and *MYHC2a*, along with the suppression of *MYHC2b* (*P* < 0.05). At the mitochondrial level, yeast β-Glu-SeNPs preserved ultrastructural integrity and enhanced mitochondrial function, as reflected by increased ATP content, elevated mtDNA copy number, and the upregulation of mitochondrial biogenesis-related genes, including *AMPK*, *PGC-1α*, *NRF1*, and *TFAM* (*P* < 0.05). Correlation analysis, molecular docking, and co-immunoprecipitation demonstrated that SelO interacts with AMPK, supporting a SelO-dependent AMPK/PGC-1α axis that drives mitochondrial biogenesis and oxidative fiber remodeling.

**Conclusion:**

Overall, yeast β-Glu-SeNPs mitigated HS-induced muscle metabolic dysfunction and meat quality deterioration via SelO-mediated mitochondrial and myofiber reprogramming.

**Supplementary Information:**

The online version contains supplementary material available at 10.1186/s40104-026-01437-4.

## Introduction

Heat stress (HS) has emerged as a major challenge to the global poultry industry [1]. Modern broilers, which have been intensively selected for rapid growth and high breast muscle yield, are now far more susceptible to thermal stress than their ancestral lines [2, 3]. Their limited capacity for heat dissipation, due to the absence of sweat glands, full feather coverage, and a high proportion of metabolically active muscle tissue, further exacerbates heat sensitivity [4, 5]. Exposure to HS leads to reduced feed intake, impaired daily weight gain, and compromised feed efficiency, accompanied by weakened immune responses, disrupted gut microbiota, and reduced carcass yield and meat quality [6–9]. Collectively, these effects culminate in substantial economic losses for commercial poultry production. Among the various impairments in growth performance and health, the decline in meat quality is one of the most visible and economically sensitive consequences of HS [10]. Elevated body temperature and metabolic disruption accelerate postmortem glycolysis, leading to a rapid drop in muscle pH, protein denaturation, increased drip loss, and paler meat color, ultimately predisposing broiler carcasses to quality defects such as pale, soft, and exudative (PSE) meat [11–13]. In addition, HS induces oxidative damage, mitochondrial dysfunction, and energy imbalance in skeletal muscle, thereby disrupting myofiber structure and compromising muscle integrity [14, 15]. Increasing evidence indicates that HS induces myofiber atrophy, disorganization of sarcomeres, and reduced fiber diameter, all of which contribute to diminished muscle integrity and postmortem meat quality [16]. Importantly, HS has been shown to alter the composition of muscle fiber types, shifting the balance from oxidative fibers toward fast-glycolytic fibers [17, 18]. This transition favors inefficient energy metabolism, reduced mitochondrial density, and lower antioxidant capacity, thereby creating a feed-forward loop that exacerbates muscle damage. Given that muscle fiber type composition fundamentally determines meat color, pH decline, water-holding capacity, and tenderness, understanding how HS reshapes fiber phenotype is essential for developing effective nutritional strategies to preserve meat quality under environmental stressors.

Dietary selenium (Se) has gained considerable attention as an essential micronutrient capable of mitigating heat-induced physiological deterioration in poultry [19]. Se exerts its biological functions predominantly through selenoproteins, which play central roles in antioxidant defense, redox homeostasis, endocrine regulation, and mitochondrial function [20]. Under HS, the increased production of reactive oxygen species and the excessive consumption of endogenous antioxidants substantially elevate the demand for Se, often leading to a decline in selenoprotein synthesis and consequent impairment of cellular defense mechanisms [21]. Therefore, optimizing Se bioavailability is considered a promising strategy to counteract HS-induced metabolic and oxidative disturbances. Se bio-efficacy is highly dependent on its chemical form [22]. Conventional inorganic Se sources exhibit low bioavailability and relatively high toxicity, whereas organic Se is safer but may be nonspecifically incorporated into proteins, leading to potential tissue accumulation [23]. Se nanoparticles (SeNPs), a zero-valent elemental form, have gained increasing attention due to their superior absorption efficiency, lower toxicity, and enhanced biological activity [24]. However, their intrinsic tendency to aggregate and their structural instability remain major obstacles that limit broader nutritional applications [25]. Polysaccharides have emerged as ideal biocompatible stabilizers to overcome these challenges [26]. Among them, yeast β-glucan, with its highly branched β-1,3/β-1,6 backbone and abundant hydroxyl groups, provides strong steric stabilization and versatile functional modification capacity [27]. Our previous work systematically optimized the synthesis of yeast β-glucan stabilized Se nanoparticles (yeast β-Glu-SeNPs) and demonstrated that yeast β-glucan effectively prevents nanoparticle aggregation, resulting in uniformly spherical particles with excellent storage stability [28]. Importantly, in Se-deficient mice, yeast β-Glu-SeNPs showed markedly better Se absorption and bioavailability than sodium selenite, selenomethionine, or yeast-bound Se. Moreover, yeast β-Glu-SeNPs significantly upregulated key selenoproteins and activated the AMPK/PGC-1α signaling pathway, ultimately driving a shift in skeletal muscle fiber types toward a more oxidative phenotype. These findings indicated that yeast β-Glu-SeNPs possess superior bioavailability, structural stability, and regulatory potency, highlighting their strong potential as a functional nutritional strategy to counteract HS-induced muscle damage in broilers. Given that HS disrupts redox homeostasis, compromises mitochondrial integrity, and drives a shift toward glycolytic muscle fibers, we formulated a reasonable hypothesis: yeast β-Glu-SeNPs may promote the remodeling of oxidative myofibers to mitigate HS-induced deterioration in meat quality, thereby restoring metabolic flexibility and preserving skeletal msuscle integrity in broilers.

Therefore, this study aimed to evaluate the efficacy of yeast β-Glu-SeNPs in improving meat quality in broilers exposed to HS and to elucidate the underlying mechanisms, with a particular focus on Se status, mitochondrial biogenesis, and muscle fiber type remodeling. This work provides new insights into how Se-based nanomaterials restore metabolic flexibility, preserve muscle structural integrity, and counteract the detrimental consequences of thermal stress.

## Materials and methods

### Animal ethics

The experimental procedures of this study were approved by the Animal Care Committee of Hunan Agricultural University, Changsha, China (Approval Number: 2020-43).

### Preparation of yeast β-Glu-SeNPs

Yeast β-glucan (≥ 98% purity) was purchased from Nanjing Taixin Biotechnology Co., Ltd. (Nanjing, China), and sodium selenite (SS) and ascorbic acid were obtained from Sigma-Aldrich (St. Louis, MO, USA). Yeast β-Glu-SeNPs were synthesized following our previously established procedure with minor modifications [28]. Briefly, β-glucan was dissolved in ultrapure water under continuous magnetic stirring until fully hydrated. A 10 mmol/L SS solution (5 mL) was then added dropwise to the β-glucan solution while stirring, and the mixture was allowed to react for 30 min. Subsequently, 5 mL of 30 mmol/L ascorbic acid was introduced as a reducing agent to initiate nanoparticle formation. The reaction proceeded at room temperature under dark conditions to prevent oxidation, yielding a stable red-colored SeNPs suspension. The resulting yeast β-Glu-SeNP dispersion was dialyzed in a 3,500 Da molecular weight cut-off dialysis bag for 12 h, with ultrapure water replaced every 2 h to remove unreacted small molecules. Finally, the dialyzed nanoparticle solution was freeze-dried under vacuum to obtain yeast β-Glu-SeNPs in powder form. The final product contained 2.6% elemental Se, with an average particle diameter of 66.2 nm, as determined by dynamic light scattering (Zetasizer Nano ZS, Malvern Instruments, UK).

### Animals, experiment design, diet, and management

A total of 400 one-day-old male Arbor Acres Plus (AA) broiler chicks were obtained from a commercial hatchery (Hunan Shuncheng Industry Co., Ltd., Changsha, China). All birds were fed a standard basal diet from d 1 to d 21. At 21 days of age, 144 broilers with similar body weights were selected and randomly assigned to a 2 × 2 factorial arrangement, consisting of Se source (SS or yeast β-Glu-SeNPs) and thermal environment (thermoneutral or HS). Birds were allotted into four treatment groups, each with 6 replicates (one cage per replicate) and 6 birds per replicate: CON group (basal diet with SS), SeNPs group (basal diet in which SS was replaced by yeast β-Glu-SeNPs), HS group (HS + basal diet with SS), and HS-SeNPs group (HS + basal diet containing yeast β-Glu-SeNPs). For the SeNPs treatments, SS in the premix was completely replaced by yeast β-Glu-SeNPs on an equivalent elemental Se basis (0.3 mg/kg diet), ensuring identical total Se levels among treatments. The basal diet was formulated to meet or exceed the nutrient requirements recommended by the feeding standards for Chinese chickens (NY/T33–2004) [29] (Table 1).

**Table 1:** Basal diet composition (air-dry basis)

Items	Contents	
	1–21 d	22–42 d
Ingredients, %		
Corn	57.50	59.90
Soybean meal	17.04	15.69
Soybean oil	1.80	3.50
Corn gluten meal	2.50	3.80
Peanut meal	15.70	12.00
Limestone	1.12	1.10
Dicalcium phosphate	2.11	1.87
Salt	0.30	0.30
L-Lysine	0.61	0.57
Methionine	0.32	0.27
Premix^1^	1.00	1.00
Total	100.00	100.00
Nutrient levels		
Metabolizable energy^2^, MJ/kg	12.38	12.81
Crude protein^3^, %	21.50	19.50
Calcium^3^, %	1.00	0.92
Total phosphorus^3^, %	0.74	0.67
Available phosphorus^2^, %	0.47	0.41
Lys^2^, %	1.29	1.15
Met^2^, %	0.53	0.53
Met + Cys^2^, %	0.93	0.85

^1^Provided per kilogram of complete diet: For broilers aged 1–21 d: vitamin A 12,000 IU, vitamin D_3_ 3,600 IU, vitamin E 45 IU, vitamin K 2.5 mg, thiamine (B_1_) 2.4 mg, riboflavin (B_2_) 5 mg, vitamin B_6_ 2.8 mg, vitamin B_12_ 0.016 mg, nicotinic acid 42 mg, calcium pantothenate 12 mg, folic acid 1 mg, biotin 0.12 mg, choline chloride 1,300 mg, Fe 80 mg, Cu 7 mg, Mn 80 mg, Zn 85 mg, I 0.7 mg, and Se 0.3 mg. For broilers aged 22–42 d: vitamin A 9,000 IU, vitamin D_3_ 1,500 IU, vitamin E 35 IU, vitamin K 2.2 mg, B_1_ 2.3 mg, B_2_ 5 mg, vitamin B_6_ 2.4 mg, vitamin B_12_ 0.015 mg, nicotinic acid 35 mg, calcium pantothenate 10 mg, folic acid 0.7 mg, biotin 0.10 mg, choline chloride 1,000 mg, Fe 80 mg, Cu 7 mg, Mn 60 mg, Zn 80 mg, I 0.6 mg, and Se 0.3 mg

^2^As calculated value

^3^As measured value

During the starter period, all birds were managed following the Chinese Production Technique Criterion for Commercial Broiler (GB/T 19664–2005). The brooding room was preheated to 33–35 °C before chick placement, after which the environmental temperature was gradually reduced according to standard broiler management practices, reaching approximately 24 °C. Relative humidity was controlled at 60%–65% during d 1–21 and at 55%–75% thereafter. Broilers had free access to feed and water throughout the entire phase. Routine husbandry practices were followed, including daily cleaning of the poultry house, adequate ventilation, and regular disinfection to maintain optimal sanitary conditions. The behavior and health status of the birds were monitored and recorded daily. All vaccinations were administered according to standard immunization programs.

From d 22 to d 42, birds in the CON and SeNPs groups were housed under thermoneutral conditions (24 ± 2 °C; relative humidity 55%–75%). In contrast, the HS and HS-SeNPs groups were subjected to cyclic HS at 32 ± 2 °C with 50%–60% humidity for 8 h/d (09:30–17:30). During the non-HS period, the temperature for these two groups was maintained at 24 ± 2 °C. HS was generated using an electric hot-air blower (Shandong Machinery Co., Ltd., Qingzhou, China), and additional humidity was regulated using a commercial ultrasonic humidifier (PC-2804, Taizhou Paichi Machinery Co., Ltd., Taizhou, China). All broilers had ad libitum access to feed and water during the entire experimental period. Temperature and relative humidity were continuously monitored and recorded daily to ensure precise environmental control.

### Growth performance

During the experimental period, feed residue and body weight were recorded weekly. These data were used to calculate average daily feed intake (ADFI) and average daily gain (ADG). Feed conversion ratio (FCR) was subsequently calculated based on ADFI and ADG.

### Sample collection

At the end of the experiment, one bird with a body weight closest to the average of each replicate was selected for sample collection. Blood samples were obtained from the wing vein using sterile syringes. After standing at room temperature, the samples were centrifuged at 3,500 r/min for 10 min, and the resulting serum was aliquoted and stored at −80 °C for subsequent biochemical and antioxidant analyses. Following blood collection, the selected birds were humanely euthanized, and both the breast and thigh muscles were carefully excised. The left-side breast and thigh muscles were used for meat quality assessment and histomorphology analysis, including pH measurements, drip loss, shear force, muscle fiber morphology, and fiber type evaluation. The right-side breast and thigh muscles were immediately placed into sterile, enzyme-free centrifuge tubes, snap-frozen in liquid nitrogen, and subsequently stored at −80 °C until further analyses.

### Meat quality

Meat quality parameters were assessed following our previously described procedures, with minor modifications [30]. Briefly, the pH values of the breast and thigh muscles at 45 min and 24 h postmortem were measured using a calibrated pH meter (EUTHE Inc., Singapore). Muscle color (L*, a*, and b* values) was determined using a portable colorimeter (Beijing Aoyik Optoelectronic Inc., Beijing, China). Breast and thigh muscle samples were suspended at 4 °C for 24 h, gently blotted with filter paper to remove surface moisture, and then weighed to calculate drip loss using the following formula:

$$Drip\ loss\ (\%) = (initial\ weight-final\ weight) / initial\ weight\ \times 100$$

Shear force was measured using a texture analyzer to evaluate meat tenderness. Fresh breast and thigh muscle samples were cut into strips parallel to the longitudinal orientation of the muscle fibers. Each sample was subjected to three repeated measurements, and the mean value was recorded as the final shear force. Post-hoc power analysis was performed using G*Power software (version 3.1.9.7) to evaluate whether the sample size used in this study was sufficient to detect significant differences in key meat quality parameters showing significant interaction effects.

### Serum biochemical parameters

Serum biochemical indices were determined using commercial assay kits following the manufacturer’s instructions (Nanjing Jiancheng Bioengineering Institute, Nanjing, China). The activities of lactate dehydrogenase (LDH), alanine aminotransferase (ALT) and aspartate aminotransferase (AST), and the concentrations of total protein (TP), albumin (ALB), globulin (GLB), and glucose (Glu) were measured using an automatic biochemical analyzer (Hitachi, Japan).

### Antioxidant capacity

The activities of total antioxidant capacity (T-AOC), superoxide dismutase (SOD), catalase (CAT), and glutathione peroxidase (GSH-Px), as well as the concentration of malondialdehyde (MDA), were determined using commercial assay kits according to the manufacturer’s instructions (Beijing Boxbio Science & Technology Co., Ltd., Beijing, China).

### ELISA

Serum corticosterone (CORT), heat shock protein 70 (HSP70), interleukin-6 (IL-6), interleukin-1β (IL-1β), and tumor necrosis factor-α (TNF-α) levels were quantified using chicken-specific ELISA kits (ELK Biotechnology, Wuhan, China) according to the manufacturer's instructions.

### Microscopic analysis of skeletal muscle structure

Breast and thigh muscle samples were fixed in 4% paraformaldehyde for 24 h, followed by dehydration through a graded ethanol series and clearing in xylene. The tissues were then embedded in paraffin and sectioned at 10 μm slices. The sections were stained with hematoxylin and eosin (HE) following standard histological procedures. Morphological parameters were quantified using CaseViewer software (v2.4, 3D HISTECH, Hungary). For each section, three randomly selected fields were evaluated to measure myofiber diameter, cross-sectional area, and fiber density.

Skeletal muscle samples were immediately cut into approximately 1 mm^3^ tissue blocks and immersed in 2.5% glutaraldehyde at 4 °C for primary fixation. After thorough rinsing in phosphate-buffered solution, the tissues were post-fixed in 1% osmium tetroxide, followed by gradual dehydration through a graded ethanol series. The dehydrated samples were then infiltrated and embedded in epoxy resin. Ultrathin sections (70–90 nm) were obtained using an ultramicrotome and mounted onto copper grids. After staining with uranyl acetate and lead citrate, the sections were examined using a transmission electron microscope.

### Glycolysis-related measurements

The contents of glycogen (Gly) and lactate (LD), as well as the activities of hexokinase (HK), pyruvate kinase (PK), and phosphofructokinase (PFK) in the breast and thigh muscles, were quantified using commercial assay kits (Nanjing Jiancheng Bioengineering Institute, Nanjing, China). Glycolytic potential (GP) was calculated according to the classical postmortem glycolysis equation:

$$GP = 2 \times Gly + LD.$$

### Immunofluorescence staining

Skeletal muscle samples from the breast and thigh were fixed in 4% paraformaldehyde for approximately 24 h. The tissues were then cryosectioned into 8–10 μm slices and permeabilized with 0.3% Triton X-100. After blocking with 5% BSA to minimize nonspecific binding, the sections were incubated overnight at 4 °C with primary antibodies specific to target proteins. On the following day, the slides were rinsed thoroughly and exposed to fluorescence-conjugated secondary antibodies for 1 h in the dark. Nuclei were counterstained with DAPI, and the sections were mounted using an anti-fade medium. Fluorescent images were acquired using a confocal laser-scanning microscope, and quantitative analysis was performed using ImageJ software.

### ATP content and mtDNA

ATP levels in thigh muscle were quantified using a commercial ATP assay kit (Beijing Boxbio Science & Technology Co., Ltd., Beijing, China) following the manufacturer’s instructions.

Mitochondrial DNA (mtDNA) abundance was evaluated as an indicator of mitochondrial biogenesis. Total DNA was extracted from thigh muscle using a genomic DNA extraction kit (Tiangen, Beijing, China). mtDNA copy number was quantified by qPCR using primers specific for mitochondrial-encoded genes (*ND1*) and normalized to a nuclear reference gene (18S rRNA). Each 20 µL reaction contained SYBR Green Master Mix, 10 ng of DNA template, and gene-specific primers. Relative mtDNA copy number was calculated using the 2^−ΔΔCt^ method.

### Real-time quantitative PCR

Total RNA was isolated from skeletal muscle samples using Trizol reagent (Accurate Biotechnology Co., Ltd., Changsha, China) following the protocol supplied by the manufacturer. RNA concentration and purity were assessed with a NanoDrop 2000 spectrophotometer (Thermo Scientific). Subsequently, 1 µg of RNA from each sample was reverse transcribed into cDNA using the PrimeScript™ RT Reagent Kit (Accurate Biotechnology Co., Ltd., Changsha, China). Quantitative PCR was conducted using SYBR Green Master Mix (Accurate Biotechnology Co., Ltd., Changsha, China) on a StepOnePlus™ Real-Time PCR System (Thermo Scientific). Primer sequences for all target genes are provided in Table S1. Relative gene expression was normalized to β-actin and calculated using the 2^−ΔΔCt^ method.

### Western blot

Protein extracts were prepared from tissues using RIPA lysis buffer supplemented with protease and phosphatase inhibitors. Equal amounts of total protein were separated on 10% SDS polyacrylamide gels and transferred onto 0.45 µm PVDF membranes. After blocking with 5% skimmed milk for 2 h, the membranes were incubated with the appropriate primary antibodies overnight at 4 °C. On the following day, membranes were washed thoroughly and then exposed to HRP-conjugated secondary antibodies for 2 h at room temperature. Immunoreactive bands were detected using an enhanced chemiluminescence (ECL) kit (Shanghai YASE Biomedical Technology Co., Ltd., Shanghai, China) and quantified using ImageJ software. Details of the primary antibodies used in this study are provided in Table S2.

### Co-immunoprecipitation

Protein–protein interactions in the thigh muscle tissue were examined using a co-immunoprecipitation (Co-IP) assay. Briefly, equal amounts of total protein were incubated with the primary antibody against the target protein overnight at 4 °C with gentle rotation. The following day, 30 μL of Protein A/G magnetic beads (MedChemExpress, USA) were added to each sample and incubated for an additional 2 h at 4 °C. After incubation, the beads were washed three to five times with lysis buffer to remove nonspecific binding. The antigen–antibody complexes were eluted by boiling the beads in SDS loading buffer for 5 min, separated by SDS-PAGE, and analyzed by Western blotting using specific antibodies to detect interacting proteins. Normal IgG was used as a negative control to exclude nonspecific precipitation.

### Molecular docking analysis

Protein–protein docking between SelO and AMPK was performed to predict their potential interaction interface. The three-dimensional structures of the two proteins were imported into the HDOCK server for semi-flexible docking, and the top-ranked docking model was selected based on the HDOCK score. The docked complex was further processed and visualized using PyMOL (version 2.0, Schrödinger, Inc., New York, NY, USA), allowing examination of key amino acid residues at the predicted binding interface. The binding affinity of the SelO-AMPK complex was estimated using the PRODIGY web server, which calculates the predicted interaction energy and stability of the protein complex.

### Statistical analysis

All data were analyzed using SPSS 26.0 (SPSS Inc., Chicago, IL, USA). A two-way analysis of variance (ANOVA) was performed to evaluate the main effects of temperature, Se source, and their interaction. When the interaction effect was significant, Tukey’s multiple comparison test was used for post hoc analysis. Results are presented as mean ± standard error of the mean (SEM). Correlation analyses were conducted using Pearson’s correlation coefficients. Principal component analysis (PCA) was performed using Origin 2023 to explore the overall variation patterns among Se-related variables and to visualize their relative contributions to the principal components. A significance level of *P* < 0.05 was considered statistically significant.

## Results

### Yeast β-Glu-SeNPs improve the growth performance of heat-stressed broilers

As shown in Table 2, no significant differences were observed in the initial body weight of broilers among the groups (*P* > 0.05). HS significantly reduced final body weight, ADG, and ADFI, whereas the Se source significantly affected these parameters, with yeast β-Glu-SeNPs improving growth performance (*P* < 0.05). No significant interaction was observed for growth performance parameters (*P* > 0.05).

**Table 2:** Effects of yeast β-Glu-SeNPs on the growth performance of heat-stressed broilers

Items^1^	TN		HS		SEM^2^	Temperature		Se source		*P*-value		
	SS	SeNPs	SS	SeNPs		TN	HS	SS	SeNPs	*P*_T_	*P*_Se_	*P*_T×Se_
IW, g	591.95	598.61	598.62	595.11	11.06	595.28	595.27	598.62	594.87	0.118	0.105	0.442
FW, g	2080.56	2,227.22	1837.50	1945.83	158.89	2,153.89	1891.67	1959.03	2086.53	0.012	0.024	0.431
ADFI, g/d	150.70	160.91	133.19	143.25	11.11	155.80	138.22	141.94	152.08	0.021	0.007	0.096
ADG, g/d	71.36	77.55	59.00	63.56	7.71	74.46	61.28	65.18	70.56	0.031	0.012	0.477
FCR	2.11	2.08	2.36	2.24	0.13	2.09	2.34	2.24	2.16	0.089	0.401	0.806

^1^Data are presented as means (*n* = 6). *TN* Thermoneutral, *HS* Heat stress, *SS* Sodium selenite, *IW* Initial body weight, *FW* Final body weight, *ADFI* Average daily feed intake, *ADG* Average daily gain, *FCR* Feed conversion ratio

^2^*SEM* Standard error of the mean

### Yeast β-Glu-SeNPs improve serum biochemical parameters in heat-stressed broilers

As shown in Table 3, significant interactions were observed for serum ALT, AST and GSH-Px activities, and the concentrations of MDA, CORT, HSP70, IL-6, and IL-1β (*P* < 0.05). HS significantly increased serum ALT and AST activities as well as the concentrations of MDA, CORT, HSP70, IL-6, IL-1β, and TNF-α in broilers, while significantly decreasing serum GSH-Px activity (*P* < 0.05). In addition, compared with SS supplementation, yeast β-Glu-SeNPs supplementation significantly increased serum GSH-Px activity and GLB concentration, while significantly reducing ALT and AST activities and the concentrations of MDA, CORT, HSP70, IL-6, and IL-1β (*P* < 0.05). Notably, compared with the HS group, broilers in the HS-SeNPs group exhibited significantly lower serum ALT and AST activities and lower concentrations of MDA, CORT, HSP70, IL-6, and IL-1β, whereas serum GSH-Px activity was significantly increased (*P* < 0.05).

**Table 3:** Effects of yeast β-Glu-SeNPs on serum biochemical, antioxidant, and stress-related parameters in heat-stressed broilers

Items^1^	TN		HS		SEM^2^	Temperature		Se source		*P*-value		
	SS	SeNPs	SS	SeNPs		TN	HS	SS	SeNPs	*P*_T_	*P*_Se_	*P*_T×Se_
Serum biochemical parameters												
TP, g/L	36.34	36.02	34.52	36.97	2.97	36.18	35.74	35.43	36.49	0.728	0.403	0.277
GLB, g/L	24.33	35.50	22.65	35.85	6.61	29.91	29.24	23.49	35.67	0.490	< 0.001	0.294
ALB, g/L	22.02	23.02	21.71	23.62	1.44	22.52	22.66	21.86	23.32	0.792	0.073	0.406
ALT, U/L	17.26^c^	15.62^c^	27.67^a^	22.39^b^	4.91	16.43	24.83	22.26	19.00	0.001	< 0.001	0.025
AST, U/L	190.00^b^	158.58^c^	232.61^a^	177.14^b^	29.60	174.29	204.87	211.31	167.86	< 0.001	< 0.001	0.013
Glu, mmol/L	9.70	11.28	11.00	11.30	1.59	10.49	11.15	10.35	11.29	0.311	0.153	0.323
LDH, U/L	463.50	440.68	525.76	450.37	28.71	452.59	488.07	494.63	445.51	0.087	0.667	0.138
Antioxidant capacity												
T-AOC, µmol/mL	0.67	0.65	0.65	0.74	0.08	0.66	0.70	0.66	0.70	0.282	0.261	0.098
CAT, U/mL	16.25	17.36	16.60	16.88	1.57	16.81	16.74	16.43	17.11	0.920	0.313	0.540
SOD, U/mL	133.71	133.74	134.87	133.95	8.06	133.72	134.41	134.29	133.84	0.113	0.295	0.270
GSH-Px, U/mL	350.93^b^	449.23^a^	263.01^c^	364.06^b^	71.46	400.08	313.53	306. 97	406.65	< 0.001	< 0.001	0.006
MDA, nmol/mL	3.61^b^	2.89^c^	6.00^a^	3.54^b^	1.28	3.24	4.77	4.80	3.21	< 0.001	< 0.001	< 0.001
Stress-related biomarkers												
CORT, ng/mL	14.05^b^	10.68^c^	26.23^a^	16.15^b^	3.13	12.37	21.19	20.14	13.42	0.012	0.001	< 0.001
HSP70, ng/mL	16.01^b^	15.44^b^	28.06^a^	17.76^b^	5.92	15.72	22.91	22.04	16.60	0.001	0.039	0.001
IL-6, pg/mL	58.72^c^	58.42^c^	156.68^a^	88.86^b^	12.68	58.57	122.77	107.70	73.64	0.009	< 0.001	< 0.001
IL-1β, pg/mL	53.77^c^	58.84^c^	99.34^a^	62.82^b^	12.33	56.31	81.08	76.56	60.83	0.021	0.006	< 0.001
TNF-α, pg/mL	21.24	19.74	56.58	31.79	10.68	20.49	44.19	38.91	24.77	0.019	0.086	0.155

^1^Data are presented as means (*n* = 6). *TN* Thermoneutral, *HS* Heat stress, *SS* Sodium selenite, *TP* Total protein, *GLB* Globulin, *ALB* Albumin, *ALT* Alanine aminotransferase, *AST* Aspartate aminotransferase, *GLU* Glucose, *LDH* Lactate dehydrogenase, *T-AOC* Total antioxidant capacity, *CAT* Catalase, *SOD* Superoxide dismutase, *GSH-Px* Glutathione peroxidase, *MDA* Malondialdehyde, *CORT* Corticosterone, *HSP70* Heat shock protein 70, *IL-6* Interleukin-6, *IL-1β* Interleukin-1β, *TNF-α* Tumor necrosis factor-α

^2^*SEM* Standard error of the mean

^a–^^c^ Values in the same row with different letters are significantly different (*P* < 0.05)

### Yeast β-Glu-SeNPs improve meat quality in heat-stressed broilers

pH, color, and water-holding capacity are key indicators of meat quality. As shown in Table 4, in breast muscle, significant interactions were observed for pH_24h_, L* and shear force (*P* < 0.05). HS significantly decreased pH_24h_ and a* value, while significantly increasing L* value, drip loss, and shear force (*P* < 0.05). In addition, compared with the SS supplementation, yeast β-Glu-SeNPs supplementation significantly increased pH_24h_, while significantly reducing L* value, drip loss, and shear force (*P* < 0.05). Notably, compared with the HS group, broilers in the HS-SeNPs group exhibited significantly higher pH_24h_ and significantly lower L* value and shear force (*P* < 0.05). In thigh muscle, significant interactions were observed for pH_45min_ and pH_24h_ (*P* < 0.05). HS significantly decreased pH_45min_, pH_24h_, and a* value, while significantly increasing L* value, drip loss, and shear force (*P* < 0.05). Compared with the SS supplementation, yeast β-Glu-SeNPs supplementation significantly increased pH_24h_ and a* value, while significantly decreasing L* value, drip loss, and shear force (*P* < 0.05). Moreover, compared with the HS group, broilers in the HS-SeNPs group showed significantly higher pH_45min_ and pH_24h_. The statistical power ranged from 0.88 to 0.99, indicating adequate statistical sensitivity for the present experimental design (Table S3).

**Table 4:** Effects of yeast β-Glu-SeNPs on meat quality parameters of heat-stressed broilers

Item^1^	TN		HS		SEM^2^	Temperature		Se source		*P*-value		
	SS	SeNPs	SS	SeNPs		TN	HS	SS	SeNPs	*P*_T_	*P*_Se_	*P*_T×Se_
Breast muscle												
pH_45min_	6.15	6.16	5.96	5.95	0.11	6.16	5.96	6.06	6.06	0.058	0.819	0.819
pH_24h_	5.84^a^	5.96^a^	5.64^b^	5.83^a^	0.12	5.90	5.73	5.74	5.90	0.001	0.001	0.003
a*	4.38	4.69	3.97	3.72	0.72	4.54	3.83	4.18	4.21	0.019	0.913	0.319
b*	7.79	7.07	7.35	7.00	0.73	7.43	7.18	7.57	7.04	0.391	0.082	0.543
L*	52.42^b^	47.71^c^	57.83^a^	50.00^b^	4.11	50.07	53.92	55.12	48.86	0.001	< 0.001	0.026
Drip loss, %	6.33	5.98	8.94	7.71	1.26	6.16	8.32	7.63	6.85	0.013	0.022	0.349
Shear force, N	34.61^b^	31.84^c^	37.10^a^	33.03^b^	2.53	33.23	35.06	35.85	32.43	0.019	0.011	0.021
Thigh muscle												
pH_45min_	6.46^a^	6.43^a^	6.21^b^	6.34^a^	0.11	6.44	6.28	6.34	6.39	0.001	0.093	0.014
pH_24h_	6.11^a^	6.32^a^	5.96^b^	6.29^a^	0.15	6.23	6.13	6.04	6.31	< 0.001	< 0.001	0.006
a*	4.45	5.67	4.15	4.98	0.83	5.06	4.57	4.30	5.33	0.021	0.001	0.460
b*	8.68	8.71	8.65	8.54	0.54	8.70	8.59	8.67	8.22	0.663	0.857	0.771
L*	56.65	47.73	60.66	51.80	5.21	52.19	56.23	58.65	49.77	0.016	0.027	0.967
Drip loss, %	7.30	5.70	9.64	7.31	1.51	6.50	8.47	8.47	6.51	0.021	< 0.001	0.091
Shear force, N	27.24	20.77	32.91	23.48	5.02	24.01	28.20	30.08	22.13	0.002	0.038	0.090

^1^Data are presented as means (*n* = 6). *TN* Thermoneutral, *HS* Heat stress, *SS* Sodium selenite

^2^*SEM* Standard error of the mean

^a–^^c^ Values in the same row with different letters are significantly different (*P* < 0.05)

### Yeast β-Glu-SeNPs improve myofiber morphology and promote muscle fiber type reprogramming

**Fig. 1:**
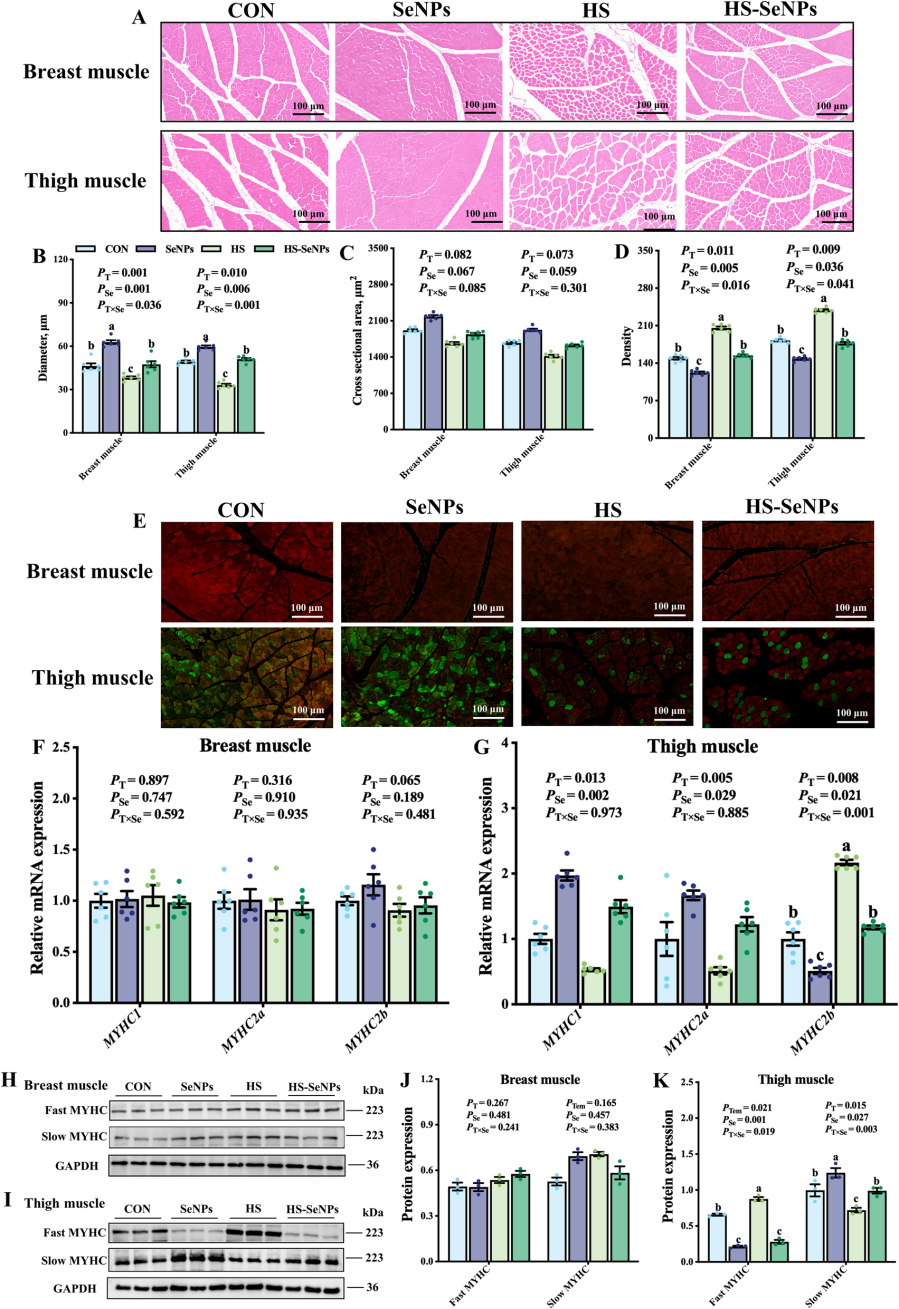
Effects of yeast β-Glu-SeNPs on myofiber morphology and fiber type composition in heat-stressed broilers. **A** Representative HE stained sections of skeletal muscle. **B** Myofiber diameter. **C** Myofiber cross-sectional area. **D** Myofiber density. **E** Immunofluorescence staining of skeletal muscle fibers. **F** and **G** Relative mRNA expression levels of muscle fiber-type marker genes. **H–K** Protein expression of fast and slow MyHC isoforms in skeletal muscle. Results are presented as mean ± SEM (*n* = 6). ^a–c^Bars with different letters differ significantly (*P* < 0.05)

As shown in Fig. 1A–D, significant interactions were observed for myofiber diameter and density (*P* < 0.05). Compared with the CON group, HS significantly decreased myofiber diameter while increasing myofiber density (*P* < 0.05). Under both thermoneutral and HS conditions, yeast β-Glu-SeNPs supplementation significantly increased myofiber diameter and reduced myofiber density (*P* < 0.05). To further evaluate muscle fiber composition, MYHC isoform profiles in breast and thigh muscles were analyzed (Fig. 1E–K). In glycolytic muscle, neither temperature nor Se source significantly affected muscle fiber type distribution (*P* > 0.05). However, in mixed muscle, significant interactions were observed for *MYHC2b* mRNA expression as well as Fast MYHC and Slow MYHC protein expression (*P* < 0.05). HS promoted a shift in muscle fiber type toward a more glycolytic phenotype, as evidenced by the significant upregulation of *MYHC2b* mRNA and Fast MYHC protein expression, together with the significant downregulation of *MYHC1* and *MYHC2a* mRNA expression and Slow MYHC protein abundance (*P* < 0.05). In contrast, compared with SS supplementation, yeast β-Glu-SeNPs significantly increased the expression of *MYHC1*, *MYHC2a*, and Slow MYHC, while reducing *MYHC2b* and Fast MYHC expression (*P* < 0.05). Moreover, compared with the HS group, broilers in the HS-SeNPs group exhibited significantly lower *MYHC2b* and Fast MYHC expression, but significantly higher Slow MYHC expression in thigh muscle (*P* < 0.05).

### Yeast β-Glu-SeNPs improve Se status in the skeletal muscle of heat-stressed broilers

**Fig. 2:**
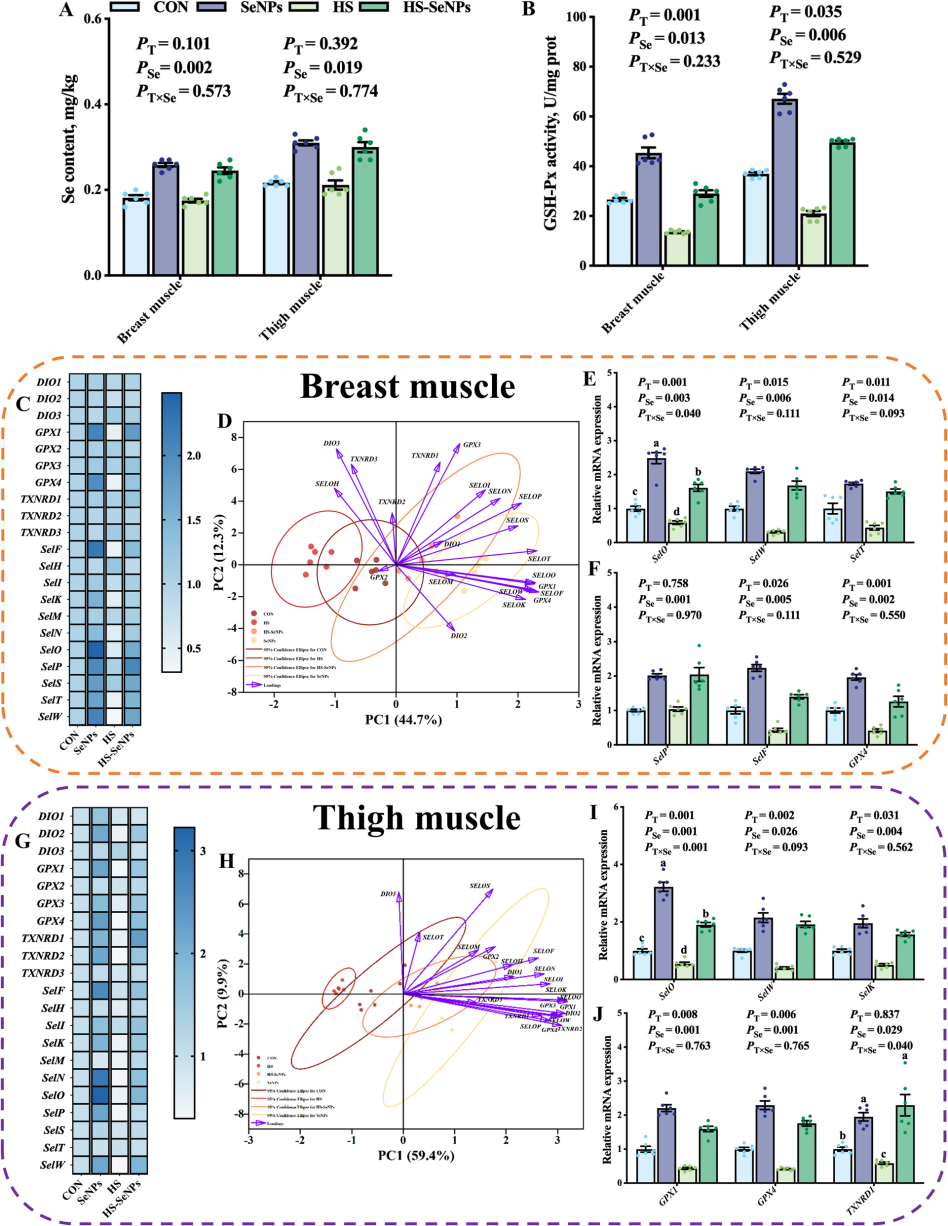
Effects of yeast β-Glu-SeNPs on Se content, GSH-Px activity, and selenoprotein transcription profiles in skeletal muscle of heat-stressed broilers. **A** Se content in skeletal muscle tissues. **B** GSH-Px activity in skeletal muscle. **C** Heatmap visualization of selenoprotein mRNA expression profiles in breast muscle. **D** PCA of breast muscle selenotranscriptome. **E** and **F** Relative mRNA expression levels of 6 key selenoproteins identified in breast muscle. **G** Heatmap visualization of selenoprotein mRNA expression profiles in thigh muscle. **H** PCA of thigh muscle selenotranscriptome. **I** and **J** Relative mRNA expression levels of 6 key selenoproteins identified in thigh muscle. Results are presented as mean ± SEM (*n* = 6). ^a–d^Bars with different letters differ significantly (*P* < 0.05)

To evaluate the Se status of skeletal muscle, total Se content (Fig. 2A), GSH-Px activity (Fig. 2B), and the mRNA expression profiles of major selenoproteins (Fig. 2C–J) were determined. HS significantly decreased GSH-Px activity in skeletal muscle (*P* < 0.05). In addition, compared with SS supplementation, yeast β-Glu-SeNPs significantly increased Se content and GSH-Px activity (*P* < 0.05). To further characterize the expression patterns of selenoproteins in skeletal muscle, PCA was performed based on the mRNA expression profiles of major selenoproteins. In breast muscle, several selenoproteins, including *SelO*, *SelW*, *SelT*, *SelP*, *SelF*, and *GPX4*, showed relatively high loadings along the PC1 axis, indicating that these genes contributed substantially to the overall variation in selenoprotein expression. Similarly, in thigh muscle, *SelO*, *SelW*, *SelK*, *GPX1*, *GPX4*, and *TXNRD1* showed relatively high contributions to the principal components. Notably, among these representative selenoproteins, *SelO* exhibited a significant interaction in both muscle types (*P* < 0.05), suggesting that it may play an important role in the regulation of skeletal muscle Se status.

### Yeast β-Glu-SeNPs modulate postmortem glycolysis in the skeletal muscle of heat-stressed broilers

**Fig. 3:**
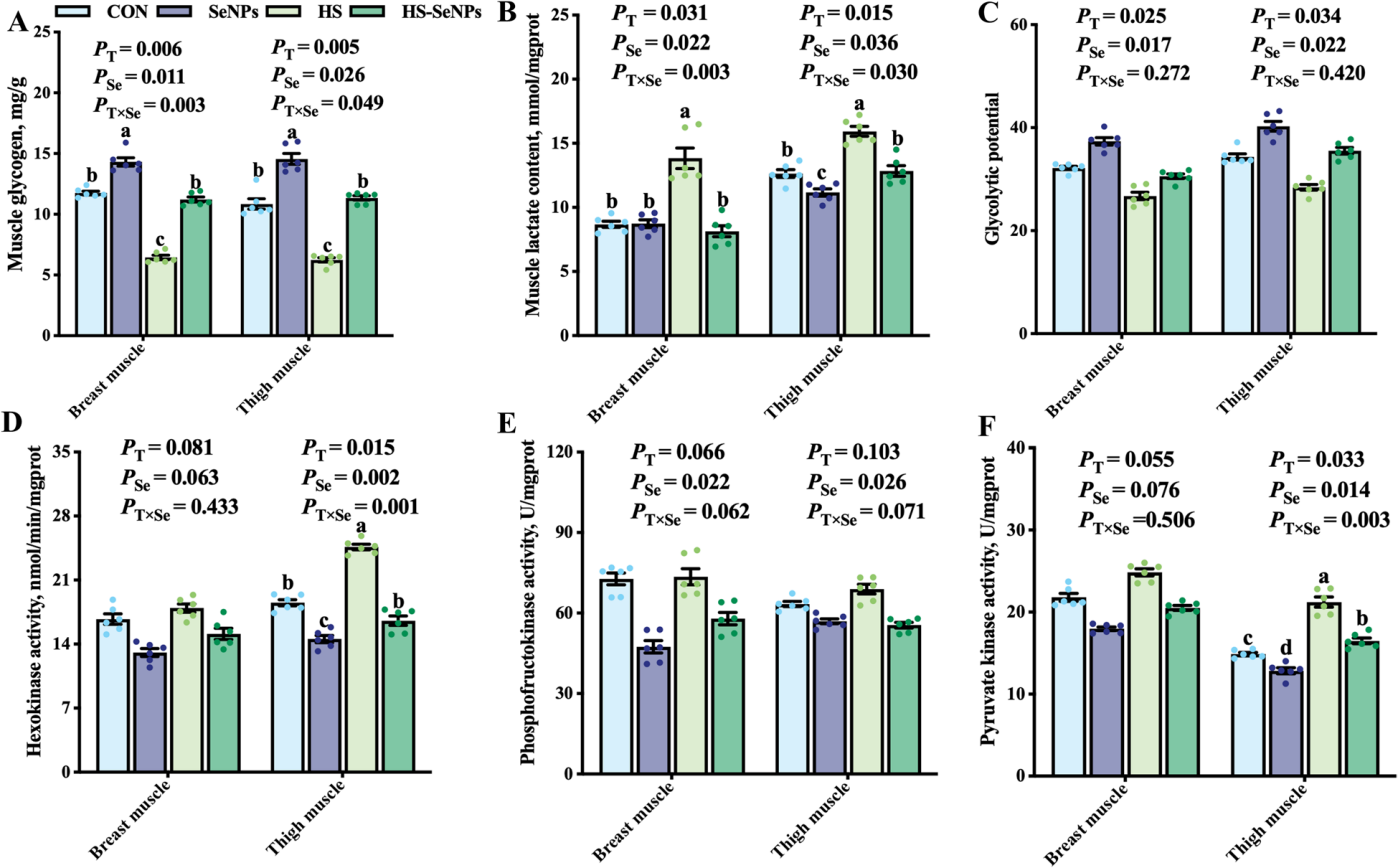
Effects of yeast β-Glu-SeNPs on postmortem glycolysis and glycolytic enzyme activities in the skeletal muscle of heat-stressed broilers. **A** Glycogen content. **B** Lactate concentration. **C** Glycolytic potential. **D** Hexokinase activity. **E** Phosphofructokinase activity. **F** Pyruvate kinase activity. Results are presented as mean ± SEM (*n* = 6). ^a–^^d^Bars with different letters differ significantly (*P* < 0.05)

Postmortem glycolytic parameters are shown in Fig. 3. In breast muscle, significant interactions were observed for glycogen and lactate contents (*P* < 0.05). HS significantly increased lactate content while decreasing glycogen content and GP (*P* < 0.05). Compared with SS supplementation, yeast β-Glu-SeNPs significantly increased glycogen content and GP, while significantly reducing lactate content and PFK activity (*P* < 0.05). Moreover, compared with the HS group, broilers in the HS-SeNPs group exhibited significantly higher glycogen content and lower lactate content (*P* < 0.05). In thigh muscle, significant interactions were observed for glycogen and lactate contents as well as HK and PK activities (*P* < 0.05). HS significantly increased lactate content and the activities of HK and PK, while significantly decreasing glycogen content and GP (*P* < 0.05). Compared with SS supplementation, yeast β-Glu-SeNPs significantly reduced lactate content and the activities of HK, PFK, and PK, while significantly increasing glycogen content and GP (*P* < 0.05). Notably, compared with the HS group, broilers in the HS-SeNPs group showed significantly higher glycogen content and significantly lower lactate content as well as HK and PK activities in thigh muscle (*P* < 0.05).

### Yeast β-Glu-SeNPs restore mitochondrial structure and function in the thigh muscle of heat-stressed broilers

**Fig. 4:**
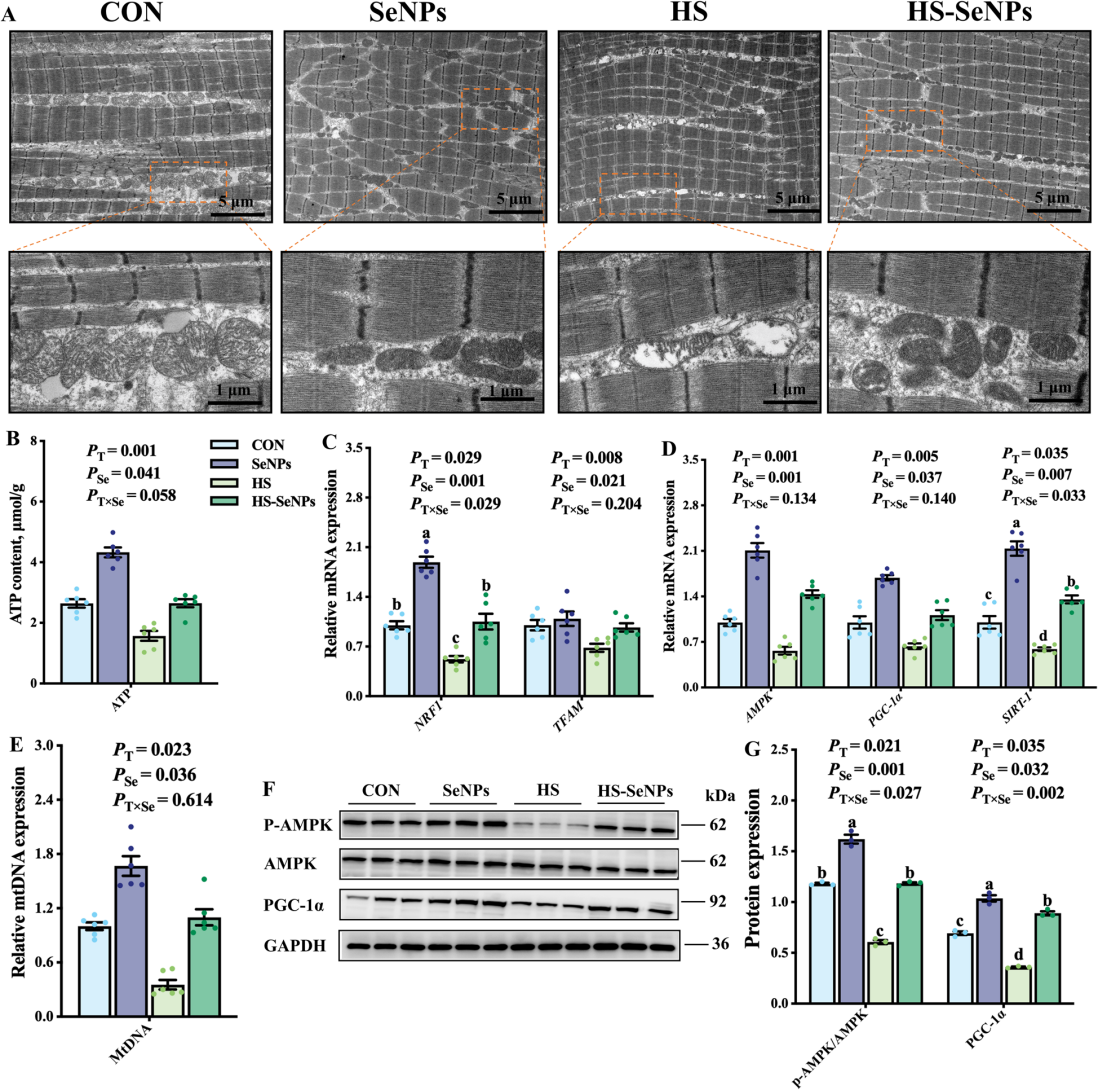
Effects of yeast β-Glu-SeNPs on mitochondrial biogenesis in the thigh muscle of heat-stressed broilers. **A** Representative transmission electron microscopy images of mitochondria in thigh muscle. **B** ATP content. **C** and **D** The mRNA expression of genes related to mitochondrial biogenesis in thigh muscle. **E** The mtDNA copy number in thigh muscle. **F** and **G** Protein expression levels of the AMPK/PGC-1α signaling pathway in thigh muscle. Results are presented as mean ± SEM (*n* = 6 or 3). ^a–^^d^Bars with different letters differ significantly (*P* < 0.05)

Transmission electron microscopy images are shown in Fig. 4A. In the CON and SeNPs groups, myofibrils and mitochondria were structurally intact. In contrast, HS induced marked mitochondrial damage, characterized by swelling, vacuolization, disrupted cristae, and disordered sarcomeres. These ultrastructural abnormalities were partially alleviated in the HS-SeNPs group. In addition, significant interactions were observed for the mRNA expression levels of *NRF1* and *SIRT-1* as well as the protein expression levels of p-AMPK and PGC-1α (*P* < 0.05). HS significantly decreased ATP content, the mRNA expression levels of *NRF1*, *TFAM*, *AMPK*, *PGC-1α*, and *SIRT-1*, mtDNA copy number, and the protein expression levels of p-AMPK and PGC-1α (*P* < 0.05). In contrast, compared with the HS group, broilers in the HS-SeNPs group exhibited significantly higher *NRF1* and *SIRT-1* mRNA expression levels as well as higher p-AMPK and PGC-1α protein expression levels in thigh muscle (*P* < 0.05).

### Yeast β-Glu-SeNPs identify SelO as a key regulatory selenoprotein linking mitochondrial biogenesis and muscle fiber remodeling

**Fig. 5:**
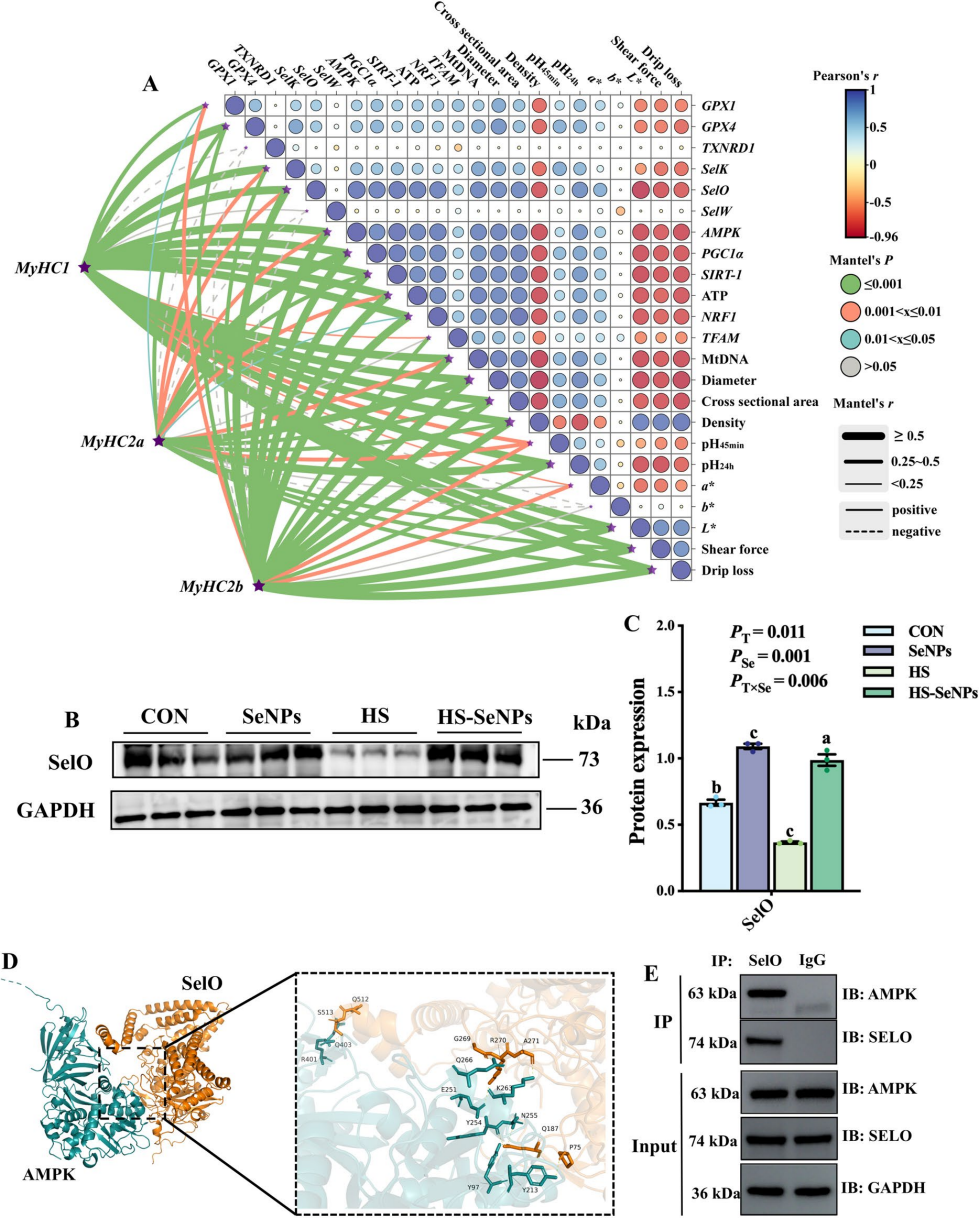
Integrated correlation analysis, SelO protein validation, molecular docking, and co-immunoprecipitation suggest that SelO is associated with mitochondrial biogenesis and muscle fiber remodeling in heat-stressed broilers. **A** Correlation network integrating meat quality traits, postmortem glycolysis parameters, mitochondrial biogenesis markers, and selenoprotein expression profiles. **B** and **C** Protein expression levels of SelO in the thigh muscle determined by western blot analysis. **D** Molecular docking model illustrating the predicted interaction between SelO and AMPK. **E** Co-IP assay confirming the physical association between SelO and AMPK in thigh muscle tissues. Results are presented as mean ± SEM (*n* = 3). ^a–^^c^Bars with different letters differ significantly (*P* < 0.05)

By integrating meat quality traits, glycolytic metabolites, mitochondrial biogenesis markers, and selenoprotein expression profiles, the correlation analysis revealed a strong functional association between the oxidative muscle fiber phenotype and several selenoproteins (Fig. 5A). Notably, *SelO* exhibited robust positive correlations with PGC-1α, *TFAM*, mtDNA copy number, ATP content, and the proportion of slow oxidative fibers, whereas it showed negative correlations with lactate accumulation, drip loss, and shear force. These patterns indicate that *SelO*, together with related mitochondrial selenoproteins, serves as a key determinant of redox homeostasis, mitochondrial integrity, and muscle fiber type reprogramming under HS. To validate SelO as a candidate regulatory selenoprotein, western blot analysis was performed (Fig. 5 B and C). HS markedly decreased SelO protein abundance in the skeletal muscle (*P* < 0.05). In contrast, yeast β-Glu-SeNPs significantly restored and further enhanced SelO expression under both normal and heat-stress conditions (*P* < 0.05), with a pronounced interaction between HS and yeast β-Glu-SeNPs treatment (*P* < 0.05). Docking simulation revealed a stable binding interface between SelO and the catalytic domain of AMPK, with multiple hydrogen bonds and hydrophobic interactions forming a structurally favorable SelO-AMPK complex (Fig. 5D). The predicted interaction was experimentally confirmed by Co-IP (Fig. 5E). SelO successfully co-precipitated with AMPK, demonstrating a direct physical association between the two proteins in skeletal muscle.

## Discussion

Growth performance is a direct reflection of the physiological and metabolic status of broilers and is among the most economically relevant outcomes in commercial production [31]. In the present study, HS markedly reduced final body weight, ADG, and ADFI, confirming the suppressive effects of thermal load on growth. Importantly, dietary supplementation with yeast β-Glu-SeNPs significantly improved body weight gain and feed intake in both thermoneutral and heat-stressed birds, indicating a potent growth-promoting and protective role. However, no significant interaction was observed, suggesting that the effects of yeast β-Glu-SeNPs on these parameters were not dependent on temperature conditions. These findings agree with earlier reports showing that HS decreases feed intake and growth efficiency by disrupting hypothalamic appetite regulation, altering intestinal health, and increasing metabolic heat production [32]. Studies using organic Se or SeNPs have shown growth-enhancing effects, but their efficacy varies due to differences in nano-formulation, stability, and Se bioavailability [33]. Compared with traditional Se sources, yeast β-Glu-stabilized SeNPs appear to provide a more consistent improvement in growth performance, likely due to their superior absorption and redox activity, as previously demonstrated in Se-deficient mice [28].

Serum biochemical parameters serve as sensitive indicators of metabolic homeostasis, hepatic integrity, oxidative stress, and systemic stress responses in poultry [34]. Enzymes such as ALT and AST reflect hepatocellular damage and protein turnover disturbances, while GSH-Px and MDA provide an overview of antioxidant defense capacity and lipid peroxidation [35]. Stress-related hormones and cytokines, including CORT, HSP70, IL-6, IL-1β, and TNF-α, directly mirror hypothalamic–pituitary–adrenal (HPA) axis activation and inflammatory burden. In the present study, HS markedly elevated serum ALT and AST activities, reduced GSH-Px activity, increased MDA accumulation, and triggered a pronounced rise in CORT, HSP70, IL-6, IL-1β, and TNF-α levels. These patterns clearly indicate hepatocellular strain, impaired antioxidant defenses, and activation of systemic stress and inflammatory pathways. Notably, dietary supplementation with yeast β-Glu-SeNPs significantly reversed these alterations under both thermoneutral and heat-stress conditions. The nanoparticles enhanced antioxidant capacity, mitigated lipid peroxidation, stabilized hepatic enzyme profiles, and markedly suppressed HPA-axis activation and inflammatory cytokine release. Our findings align with previous studies reporting that HS disrupts hepatic function and elevates oxidative damage in broilers [36]. Similar improvements in antioxidant enzymes and reductions in inflammatory mediators have been described following supplementation with organic Se, polysaccharides, or Se nanoparticles preparations [37–39]. In the present study, yeast β-glucan primarily served as a stabilizing matrix during the synthesis of Se nanoparticles rather than as an independent dietary component. Therefore, the observed effects are interpreted primarily as resulting from the stabilized Se nanoparticles, although the potential contribution of the polysaccharide matrix cannot be completely excluded. The strong anti-inflammatory effects observed here are also consistent with recent Se nanoparticles research highlighting enhanced immunomodulatory capacity, but our results uniquely demonstrate pronounced suppression of CORT and HSP70, indicating deeper regulation of the neuroendocrine stress axis [40]. The protective effects of yeast β-Glu-SeNPs likely stem from multiple interrelated mechanisms. Owing to their nanoscale structure, yeast β-Glu-SeNPs exhibit enhanced intestinal absorption and are more efficiently incorporated into selenoproteins, thereby strengthening ROS-scavenging capacity and limiting lipid peroxidation, as reflected by increased GSH-Px activity and reduced MDA levels [41]. The resulting attenuation of oxidative damage alleviates hepatic metabolic burden, contributing to the normalization of serum ALT and AST activities [42]. In addition, adequate Se availability has been shown to modulate stress responsiveness by dampening HPA-axis overactivation, which may underlie the reduced CORT secretion and HSP70 accumulation observed under HS conditions [43]. Concurrently, nano-Se-mediated suppression of NF-κB signaling likely accounts for the decreased circulating pro-inflammatory cytokines [44]. Taken together, these mechanisms converge to restore systemic homeostasis and counteract the metabolic and inflammatory burden imposed by HS.

Meat quality parameters, including ultimate pH, color attributes, water-holding capacity, and tenderness, are central determinants of consumer acceptance and commercial value [45]. Key traits such as pH, meat color, water-holding capacity, and tenderness are tightly regulated by postmortem glycolytic kinetics, muscle fiber composition, and intracellular oxidation status [46]. In the present study, HS markedly impaired meat quality, as indicated by reduced pH_24h_, increased L* values, and elevated drip loss and shear force in both breast and thigh muscles. Importantly, dietary supplementation with yeast β-Glu-SeNPs alleviated these deficits by increasing pH_24h_, reducing lightness, improving water-holding capacity, and lowering shear force. Our findings are consistent with earlier reports showing that Se supplementation improves meat quality by enhancing antioxidative capacity and stabilizing muscle proteins under thermal or oxidative stress conditions. Compared with conventional Se sources, SeNPs may exhibit higher bioavailability, greater surface activity, and improved cellular uptake, which could facilitate more efficient incorporation into selenoproteins involved in redox regulation and thereby contribute to reduced heat-induced oxidative damage, improved mitochondrial function and energy metabolism, and better muscle integrity and meat quality under HS conditions [47, 48]. These outcomes demonstrate that β-Glu-SeNPs can partially restore postmortem muscle metabolic stability, thereby improving the sensory and functional attributes of heat-stressed broiler meat.

In this study, HS markedly reduced muscle fiber diameter and increased fiber density in both breast and thigh muscles, suggesting fiber shrinkage and intracellular water loss, a typical morphological hallmark of thermal injury. Moreover, HS promoted a shift from oxidative toward glycolytic fibers, as demonstrated by decreased *MyHC1* and *MyHC2a* transcripts and proteins and elevated Fast-MyHC expression. Importantly, dietary yeast β-Glu-SeNPs effectively rescued these morphological damages, increasing fiber diameter and decreasing density, while simultaneously enhancing oxidative fiber markers and suppressing glycolytic *MyHC2b* expression. These results indicate that yeast β-Glu-SeNPs promote oxidative fiber remodeling under both normal and heat-stressed conditions. Previous studies have reported that HS reduces muscle fiber size due to protein breakdown and cellular dehydration, and those oxidative fibers are more sensitive to thermal metabolic disruptions [49, 50]. Our observations align with reports showing that acute or chronic HS downregulates oxidative MyHC isoforms and upregulates glycolytic types, thereby aggravating PSE-like meat quality. Notably, most previous Se-related studies focused on Se-Met or sodium selenite, which rarely demonstrated significant remodeling of muscle fiber types. Recent work with nano-Se suggested potential benefits for mitochondrial integrity, but few studies have directly linked SeNPs to fiber-type transitions [51, 52]. Thus, our findings extend the current understanding by showing that yeast β-Glu-SeNPs possess a unique capacity to stabilize muscle structure and promote oxidative fiber phenotypes under HS.

Se status is a fundamental determinant of redox homeostasis, mitochondrial integrity, and muscle metabolic stability in poultry [53]. As an essential micronutrient, Se is incorporated into at least 25 selenoproteins, many of which function as antioxidant enzymes or endoplasmic reticulum-associated redox regulators [54]. These proteins are indispensable for maintaining muscle redox balance, safeguarding mitochondrial structure, and supporting normal myofiber phenotype. In the present study, HS markedly reduced GSH-Px activity in both breast and thigh muscles, indicating compromised Se utilization and impaired enzymatic antioxidant defense. Supplementation with yeast β-Glu-SeNPs effectively reversed these deficits, as evidenced by significantly increased Se accumulation and enhanced GSH-Px activity regardless of ambient conditions. PCA-based profiling further highlighted a subset of selenoproteins, including *SelO*, *SelW*, *SelT*, *SelP*, *SelF*, and *GPX4* in breast muscle and *SelO*, *SelW*, *SelK*, *GPX1*, *GPX4*, and *TXNRD1* in thigh muscle, which showed relatively higher contributions to the principal components associated with muscle Se status. The observed reduction in Se deposition and GSH-Px activity under HS aligns with earlier reports demonstrating that thermal stress accelerates oxidative consumption of Se-dependent enzymes and limits their synthesis due to increased ROS load [55]. However, yeast β-Glu-SeNP supplementation in the present study markedly improved Se deposition and selenoprotein-related parameters in skeletal muscle. These findings are consistent with previous reports indicating that polysaccharide-stabilized Se nanoparticles exhibit higher bioavailability and stronger regulatory effects on muscle selenoproteins compared with conventional Se sources [28, 56].

Glycolytic potential reflects the total substrate reservoir available for postmortem glycolysis, primarily determined by glycogen content and lactate accumulation [57]. It directly governs the rate and extent of pH decline after slaughter, thereby influencing protein denaturation, water-holding capacity, meat color, and overall meat quality [58]. In this experiment, HS markedly reduced glycogen reserves and GP in both breast and thigh muscles, while simultaneously elevating lactate levels and upregulating key glycolytic enzymes. These changes indicate a state of intensified pre-slaughter glycolysis and accelerated depletion of metabolic substrates, consistent with a shift toward anaerobic metabolism under thermal strain. Supplementation with yeast β-Glu-SeNPs effectively counteracted these disruptions by restoring glycogen content, lowering lactate accumulation, and reducing glycolytic enzyme activity, thereby elevating overall GP in both muscle types. These findings suggest that SeNPs maintain a more balanced postmortem metabolic trajectory, preventing excessive glycolytic acceleration and thus supporting better meat quality. The reduction in GP under HS likely arises from heightened metabolic demand, impaired mitochondrial ATP synthesis, and enhanced reliance on anaerobic glycolysis [59]. Increased HK and PK activities may reflect a compensatory metabolic response to mitochondrial dysfunction under HS conditions [60]. These responses may help maintain glycogen reserves and limit excessive activation of anaerobic glycolysis during postmortem metabolism. In addition, regulation of selenoproteins involved in redox homeostasis may further contribute to maintaining metabolic stability in skeletal muscle under HS conditions.

Mitochondrial biogenesis is a central determinant of skeletal muscle metabolic flexibility, oxidative capacity, and resilience under environmental stress [61]. Adequate mitochondrial quantity and function are essential for maintaining ATP production, controlling reactive oxygen species, and supporting oxidative myofiber phenotype stability [62]. Disruptions in mitochondrial biogenesis, particularly downregulation of key regulators such as PGC-1α, NRF1, and TFAM, are strongly associated with impaired muscle function, increased glycolytic shift, and reduced meat quality in heat-stressed broilers [63]. In the present study, HS markedly suppressed mitochondrial biogenesis in broiler thigh muscle, as shown by reduced ATP content, decreased mtDNA copy number, and downregulation of PGC-1α and TFAM expression, together with ultrastructural defects captured by TEM. These changes were accompanied by a shift toward glycolytic fiber types and deteriorated meat quality. Importantly, supplementation with yeast β-Glu-SeNPs restored mitochondrial biogenesis, elevating ATP levels, mtDNA abundance, and oxidative fiber markers, while repairing cristae damage. PCA-based screening and correlation analysis further identified SelO as a key Se-responsive factor tightly associated with mitochondrial function, showing strong positive correlations with PGC-1α, TFAM, oxidative myofiber genes, and ATP content. These findings are consistent with recent studies, which highlight SelO as an essential mitochondrial Ubi-GCN5-like protein involved in AMPylation, redox relay maintenance, and mitochondrial proteases [64, 65]. To date, however, few studies have experimentally validated the direct interaction between SelO and key metabolic regulators under HS. Mechanistically, the combined evidence from molecular docking, CO-IP assays, and phenotypic correlations strongly suggests that SelO acts upstream of AMPK, enabling the activation of the AMPK-PGC-1α axis and subsequent mitochondrial biogenesis. The predicted high-affinity binding model and experimentally verified protein–protein interaction imply that SelO may modulate AMPK activation through redox-dependent AMPylation or stabilization mechanisms, thereby facilitating mitochondrial transcriptional programming. The restoration of mitochondrial biogenesis ultimately supports the preservation of oxidative fiber phenotype, prevents excessive glycolytic metabolism, and contributes to improved meat quality under HS. Thus, yeast β-Glu-SeNPs appear to exert their protective effects through a SelO-centered regulatory network that reinforces mitochondrial integrity and muscle metabolic homeostasis.

As with most studies, the present study has certain limitations. Because the experiment was performed under controlled conditions, the overall replication level and design scope were inevitably constrained, while the absence of a yeast β-glucan-only group limited our ability to fully disentangle the independent contribution of the polysaccharide matrix from that of the selenium nanoparticles. Future studies with expanded designs and additional control groups are therefore warranted to further validate these findings and elucidate the underlying mechanisms.

## Conclusion

In summary, yeast β-Glu-SeNPs effectively alleviated HS-induced muscle impairment in broilers. Yeast β-Glu-SeNPs increased skeletal muscle Se content, enhanced GSH-Px activity, and selectively upregulated key selenoproteins, particularly SelO. The activation of SelO was closely associated with improved mitochondrial biogenesis through AMPK/PGC-1α signaling, leading to increased mtDNA copy number, ATP production, and mitochondrial structural integrity. Moreover, yeast β-Glu-SeNPs promoted a metabolic shift toward oxidative myofiber phenotypes, mitigated excessive glycolysis, and ultimately improved meat quality.

## Supplementary Information

Additional file 1: Table S1. Primer sequences for real-time quantitative PCR analysis. Table S2. Antibody Information. Table S3. Summary of post hoc power analysis based on meat quality.

Additional file 2: The full uncropped Western blots images.

## Data Availability

The datasets used and/or analysed during the current study are available from the corresponding author on reasonable request.

## Abbreviations

ADG: Average daily gain
ADFI: Average daily feed intake
ALB: Albumin
ALT: Alanine aminotransferase
AST: Aspartate aminotransferase
CAT: Catalase
CORT: Corticosterone
FCR: Feed conversion ratio
GLB: Globulin
Glu: Glucose
GLY: Glycogen
GP: Glycolytic potential
GSH-Px: Glutathione peroxidase
HK: Hexokinase
HSP70: Heat shock protein 70
IL-6: Interleukin-6
IL-1β: Interleukin-1β
LD: Lactate
LDH: Lactate dehydrogenase
MDA: Malondialdehyde
PFK: Phosphofructokinase
PK: Pyruvate kinase
SOD: Superoxide dismutase
T-AOC: Total antioxidant capacity
TNF-α: Tumor necrosis factor-α
TP: Total protein
