# Supplementary material for: Yeast β-glucan selenium nanoparticles enhance meat quality in heat-stressed broilers via SelO-mediated mitochondrial biogenesis and oxidative myofiber remodeling
Source: J Anim Sci Biotechnol. 2026 Jul 4;17:138. doi: 10.1186/s40104-026-01437-4 (PMC13332611; doi:10.1186/s40104-026-01437-4)
Supplement: Supplementary file 1 — Additional file 1: Table S1. Primer sequences for real-time quantitative PCR analysis. Table S2. Antibody Information. Table S3. Summary of post hoc power analysis based on meat quality. [file 40104_2026_1437_MOESM1_ESM.docx]

**Table S1** Primer sequences for real-time quantitative PCR analysis

| **Gene symbols** | **Production length** | **Accession No.** | **Primer sequence (5’ to3’)** |
| --- | --- | --- | --- |
| *β-actin* | 89 | NM_205518.2 | F: CCAGCCATGTATGTAGCCATCCAG  R: ACACCATCACCAGAGTCCATCACA |
| *MYHC-1* | 141 | XM_046902785.1 | F: GCTCTCAGGGCGTAACAACT  R: CAAAGTGCATGATAGCGCCC |
| *MYHC-2a* | 141 | NM_204228.4 | F: CAAGCATTGACCAGCTGCC  R: AGATAAGGAGCAGCCTCCCC |
| *MYHC-2b* | 131 | NM_001013396.2 | F: TCCAGTCAGCACAAGACCTTC  R: GACTTTCGGAGGTAGGGAGC |
| *AMPK* | 59 | NM_001039603.2 | F: CCATCTGTCTCGCCCTCATCCT  R: GCCGTGGTGCTTCAGCTACTAA |
| *PGC-1α* | 126 | NM_001006457.2 | F: AACCAGCAGAGAACAGGAACA  R: ACTCAGGTGTCAATGGAAGTGAT |
| *SIRT-1* | 140 | NM_001004767.2 | F: AGCGGCTCGTGTCACAGTCA  R: GCATCTTGTTCAACTCCATCCTCCT |
| *DIO1* | 62 | NM_001097614.2 | F: TCTACAAGGGAGGAGTGGGG  R: TTCCAGGACAGCGCGTATTT |
| *DIO2* | 137 | NM_001324555.3 | F: CGCTGTATGACTCTGTGATCCTCCT  R: TTGCTGCCATCGTTGCCCTTG |
| *DIO3* | 148 | NM_001122648.3 | F: TGTGATGCTCTGGCTCCTGGATT  R: CAGCGTGAACATGCGGTTGGA |
| *GPX1* | 166 | NM_001277853.3 | F: GCAAAGTGCTGCTGGTGGTCAA  R: ATCTCCTCGTTGGTGGCGTTCT |
| *GPX2* | 60 | NM_001277854.3 | F: ACCAGTTCGGCTACCAGGAGAA  R: CGTGCTTGAGGCAGTTGAGGAT |
| *GPX3* | 54 | NM_001163232.3 | F: CGCTGAGGAACCACGACATCAA  R: CAGTGCCCACCAGGAACTTCTC |
| *GPX4* | 152 | NM_001346448.2 | F: CGGCCACCTCCATCTACGACTT  R: GTGCAGATCGACGAGCTGAGTG |
| *TXNRD1* | 95 | NM_001030762.4 | F: TGGCAATCCAGGCAGGAAGACT  R: AGTGAACACCGTGGTGGGAACA |
| *TXNRD2* | 50 | NM_001122691.3 | F: TGGAGTGTAGCCCACCCAGTTC  R: GCTTGAGCCATCACAGACCAGT |
| *TXNRD3* | 135 | NM_001122777.3 | F: TGGTGCGTTCCATACTCCTTCG  R: GTGTGCCATCCTCCAGCCTTTC |
| *SELENOF* | 58 | NM_001012926.3 | F: AGTACGTGCGTGGTTCTGAC  R: GCAATGTTCCCACTGTCGTC |
| *SELENOH* | 162 | NM_001277865.2 | F: TCGTCATCGAGCACTGCCGTA  R: TGCTGCCGTCTTCCTTCACCA |
| *SELENOI* | 92 | NM_001031528.4 | F: GTTGGAACCGCCTTCGCTAACA  R: GGCAGCAGCATCCAGTTCAGAG |
| *SELENOK* | 93 | NM_001025441.2 | F: ATAAATCACTGGGGTGGAGGC  R: GCTCCTTTGCCTGCTTCTTAC |
| *SELENOM* | 126 | NM_001277859.2 | F: ACATCCCGCTGTACCATAACCT  R: TCTCCTCCCGGGTCATGTC |
| *SELENON* | 59 | NM_001114972.3 | F: GGCGAGTGAAGGCAGCAACAT  R: CCAGCTCCATCTGAGGCAGGTA |
| *SELENOO* | 78 | NM_001115017.5 | F: GCGGTCAAGCATACGGGAGTTC  R: CAGGTTCCAGCCCTCGTTGTTG |
| *SELENOP* | 81 | NM_001031609.3 | F: CCAAGTGGTCAGCATTCACATC  R: ATGACGACCACCCTCACGAT |
| *SELENOS* | 101 | NM_001024734.3 | F: ATGGCTGGTACATCCTCCTGGC  R: AGTTGCTCCTTGCTGGCTGCT |
| *SELENOT* | 78 | NM_001006557.4 | F: GCCGCTGCTCAAGTTCCAGATC  R: TGGCTGATGACCCGCATGTACT |
| *SELENOW* | 63 | NM_001166327.2 | F: TGACGGGATGGTTCGAGGTGAC  R: CGTCGCCGTTCTTCTTGGAGTG |
| *NRF1* | 155 | XM_015282528.4 | F: CTACCACAGTTACCGTCGCACAAG  R: TGCTGCTTCTGCTAATGATGCTACC |
| *TFAM* | 144 | NM_204100.2 | F: GACCTCGAAGTGGCTTCAAC  R: GAGCAAGCTGAAGGTATGGCT |
| *ND1* | 75 | NC_053523.1 | F: TGCCCACCCTAACAAACCTT  R: TTAAGAAGGCCACGGCGATT |
| *18s* | 123 | NC_052547.1 | F: GACACGGACAGGATTGACAGATTGA  R: ATGCCAGAGTCTCGTTCGTTATCG |

**Table S2** Antibody Information

| **Name** | **Brand** | **Source** | **Dilution ratio** |
| --- | --- | --- | --- |
| Primary antibodies SELO | ELK, Hangzhou, Chian | Rabbit Polyclonal | 1:1000 |
| Primary antibodies p-AMPK | Daige, Hangzhou, Chian | Rabbit Polyclonal | 1:1000 |
| Primary antibodies AMPK | Daige, Hangzhou, Chian | Rabbit Polyclonal | 1:1000 |
| Primary antibodies PGC-1 | Daige, Hangzhou, Chian | Rabbit Polyclonal | 1:1000 |
| Primary antibodies Slow | Servicebio, Wuhan, Chian | Rabbit Polyclonal | 1:1000 |
| Primary antibodies Fast | Servicebio, Wuhan, Chian | Rabbit Polyclonal | 1:1000 |
| Primary antibodies GAPDH | Servicebio, Wuhan, Chian | Rabbit Polyclonal | 1:1000 |
| Secondary antirabbit HRP-conjugated antibodies | Servicebio, Wuhan, Chian | Rabbit IgG | 1:10000 |

**Table S3** Summary of post hoc power analysis based on meat quality

| **Items** | **Sample size** | **Significance level (α)** | **Statistical power (1-β)** |
| --- | --- | --- | --- |
| Breast muscle | | | |
| pH_24h_ | 6 | 0.05 | 0.99 |
| *L** | 6 | 0.05 | 0.88 |
| Shear force | 6 | 0.05 | 0.90 |
| Thigh muscle | | | |
| pH_45min_ | 6 | 0.05 | 0.94 |
| pH_24h_ | 6 | 0.05 | 0.98 |
