# Supplementary figures and images for: Yeast β-glucan selenium nanoparticles enhance meat quality in heat-stressed broilers via SelO-mediated mitochondrial biogenesis and oxidative myofiber remodeling
Source: J Anim Sci Biotechnol. 2026 Jul 4;17:138. doi: 10.1186/s40104-026-01437-4 (PMC13332611; doi:10.1186/s40104-026-01437-4)

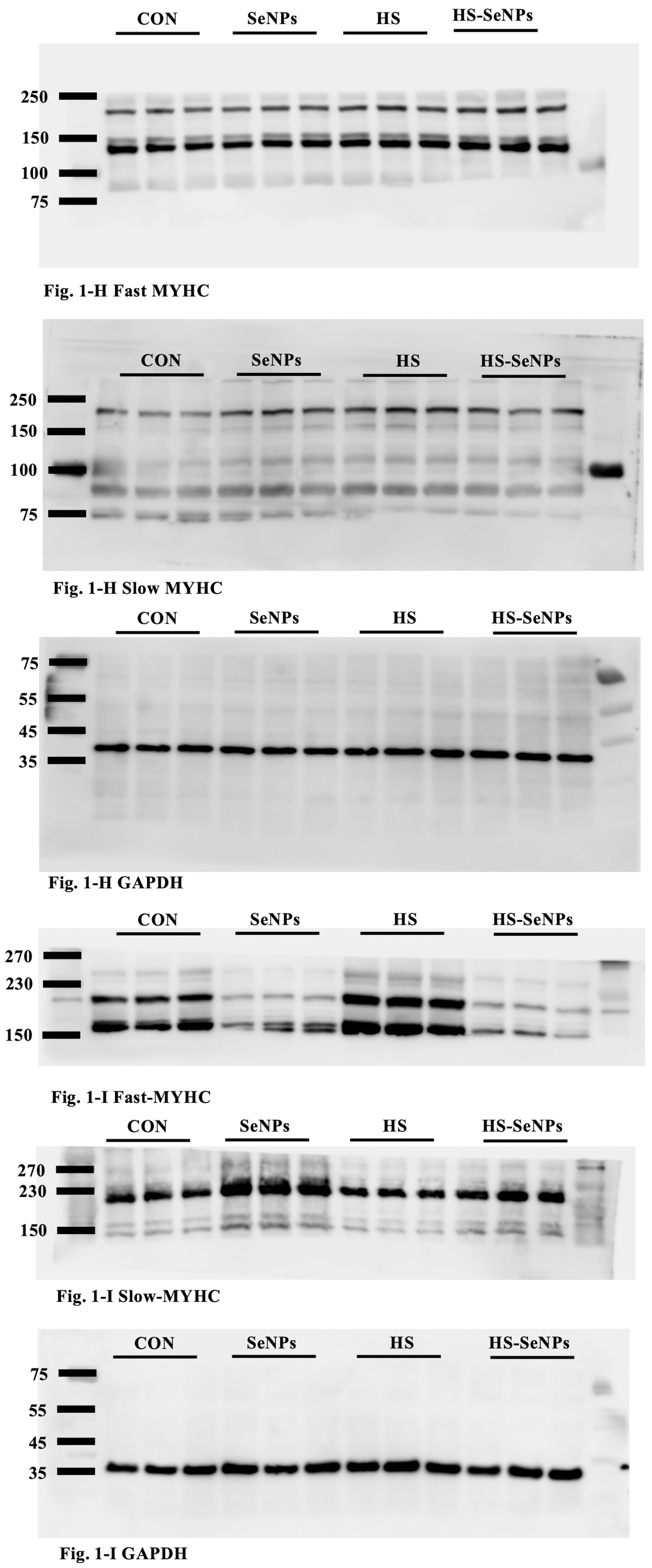


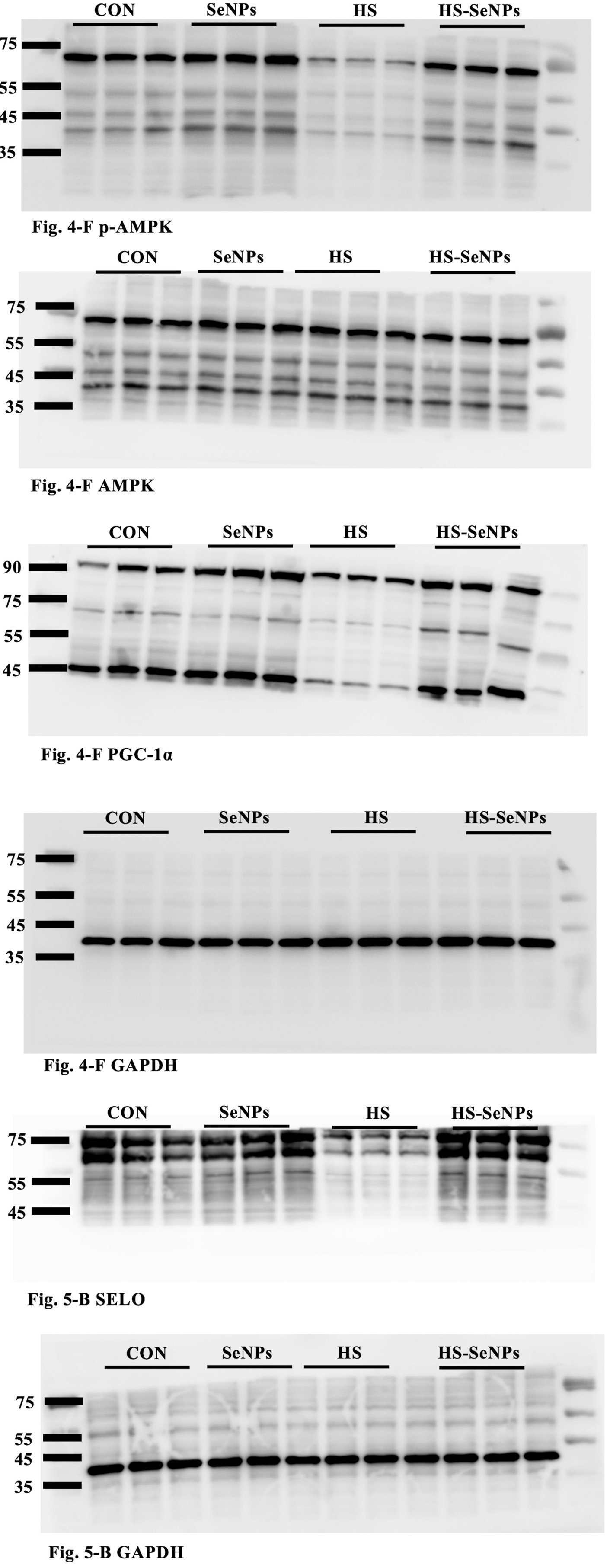


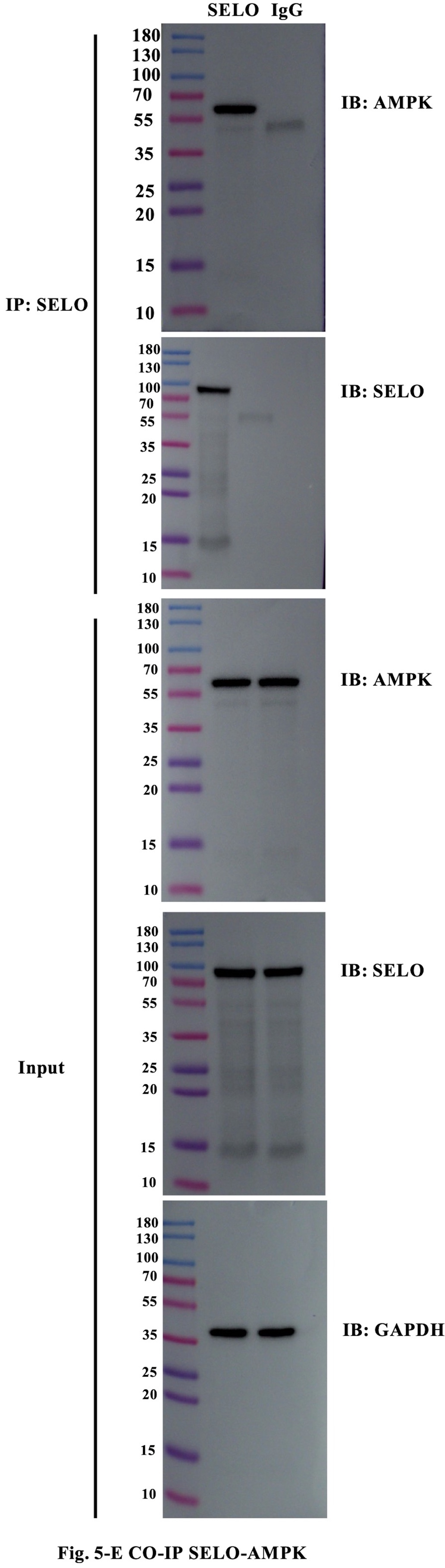

Supplement: Supplementary file 2 — Additional file 2: The full uncropped Western blots images. [file 40104_2026_1437_MOESM2_ESM.docx]
